# Coherence as resource in scattering quantum walk search on complete graph

**DOI:** 10.1038/s41598-018-29342-5

**Published:** 2018-07-23

**Authors:** Yun-Long Su, Si-Yuan Liu, Xiao-Hui Wang, Heng Fan, Wen-Li Yang

**Affiliations:** 1 Institute of Modern Physics, Northwest University, Xi’an, 710069 China; 2 School of Physics, Northwest University, Xi’an, 710069 China; 3 Shaanxi Key Laboratory for Theoretical Physics Frontiers, Xi’an, 710069 China; 4 Institute of Physics, Chinese Academy of Sciences, Beijing, 100190 China

## Abstract

We investigate the behavior of coherence in scattering quantum walk search on complete graph under the condition that the total number of vertices of the graph is significantly larger than the marked number of vertices we are searching, *N* ≫ *v*. We find that the consumption of coherence represents the increase of the success probability for the searching, also it is related to the efficiency of the algorithm in oracle queries. If no coherence is consumed or an incoherent state is utilized, the algorithm will behave as the classical blind search, implying that coherence is responsible for the speed-up in this quantum algorithm over its classical counterpart. The effect of noises, in particular of photon loss and random phase shifts, on the performance of algorithm is studied. Two types of noise are considered because they arise in the optical network used for experimental realization of scattering quantum walk. It is found that photon loss will reduce the coherence and random phase shifts will hinder the interference between the edge states, both leading to lower success probability compared with the noise-free case. We then conclude that coherence plays an essential role and is responsible for the speed-up in this quantum algorithm.

## Introduction

Random walk is an important prototype for efficient classical algorithms^1,2^. As the quantum analogy of random walk, quantum walk is important in developing efficient quantum algorithms^3^. As we have known, the quantum algorithms may have great speed-up compared with their classical counterparts as shown by Shor’s algorithm and Grover search^4,5^. There are two kinds of quantum walk, discrete quantum walk and continuous quantum walk. Both of them show their advantages over the classical random walks^6,7^. A quantum search algorithm constructed from discrete quantum walk on hypercube demonstrates similar boost over the classical search algorithms as the Grover search algorithm^8^. A search algorithm based on continuous quantum walk has similar quadratic speed-up^9^. Quantum walk is not only studied in theory but also realized in experiment via various ways^10–16^. To implement quantum walk using linear optical elements, scattering quantum walk is proposed^17^. An search algorithm based on such quantum walk is constructed and analyzed on complete graph explicitly, showing analogous quadratic speed-up^18^.

On the other hand, we know that quantum entanglement plays a significant role in quantum teleportation, super-dense coding and quantum phase transitions in many-body systems^19,20^. Also other quantum correlations, such as quantum discord, are critical in quantum information and many-body systems^21,22^. All of them can be assumed to be some kind(s) of resource(s) for quantum information processing^20,23–25^. Recently, the resource theory of quantum coherence is proposed, and has attracted much attention^26–28^. Remarkably, coherence is shown to be a valuable resource in several well-known quantum algorithms. Explicitly, it is shown that coherence is a resource in Deutsch-Jozsa algorithm based on quantum walk^29^. Also, coherence depletion is shown to be related with probability of success in Grover search algorithm^30^. Coherence is assumed as resource in deterministic quantum computation with one qubit^31^. The interference in quantum walk makes quantum walk differ from the classical random walk, so it is expected that coherence should play a key role in quantum walk algorithms. In particular, we wonder whether quantum coherence is directly responsible for the speed-up of the quantum walk search based on complete graph studied in the reference^18^.

In this paper, the role of coherence is studied systemically in a quantum walk search algorithm, the scattering quantum walk search^18^. We consider the condition that the total number of vertices *N* of the graph, which is the scope of the data, is greatly larger than the marked number of vertices *v* we are searching, $N\gg v > 1$. The quantum search algorithm demonstrates great advantage over classical ones under this condition. Two measures of coherence are used to study the dynamics of coherence^26^ and relate the searching probability of success with coherence. In the progress of the algorithm, the coherence is decreasing with the increase of probability of success. The coherence reaches its minimum when the success probability is maximal. When reducing the efficiency of the algorithm, while the minimum of coherence increases, the connection between the coherence and probability of success still exists. Besides, when there is no coherence consumed, this quantum search algorithm will have the same complexity as that of the classical blind search algorithm. If the consumption of coherence is below a proper value, the efficiency of the algorithm will be lower than classical search with memory. Furthermore, when initial states are incoherent, also $N\gg v$, the probability of finding the targets almost keeps unchanged compared to that of the start stage. So we cannot use a state with no coherence to perform the search algorithm.

The effect of the decoherence on the performance of the algorithm is also investigated. An experimental prototype for realizing scattering quantum walk search is depicted based on the optical networks and two realistic types of noises are considered, photon losses and random phase shifts. When the photon loss is considered, it leads to the decrease of the success probability along with the reduce of coherence. Random phase shifts hinder the interference between the edge states, resulting in the low success probability and large coherence. Based on those results, we conclude that the coherence plays an essential role and is responsible for the speed-up.

## Brief Introduction of Scattering Quantum Walk On Complete Graph

The quantum walk search algorithm we studied here is the scattering quantum walk search^18^. Scattering quantum walk^17^ is one of the versions of discrete quantum walk and is unitary equivalent to the coined quantum walk^32^. It could be considered as the discrete quantum walk in optical network.

**Figure 1:**
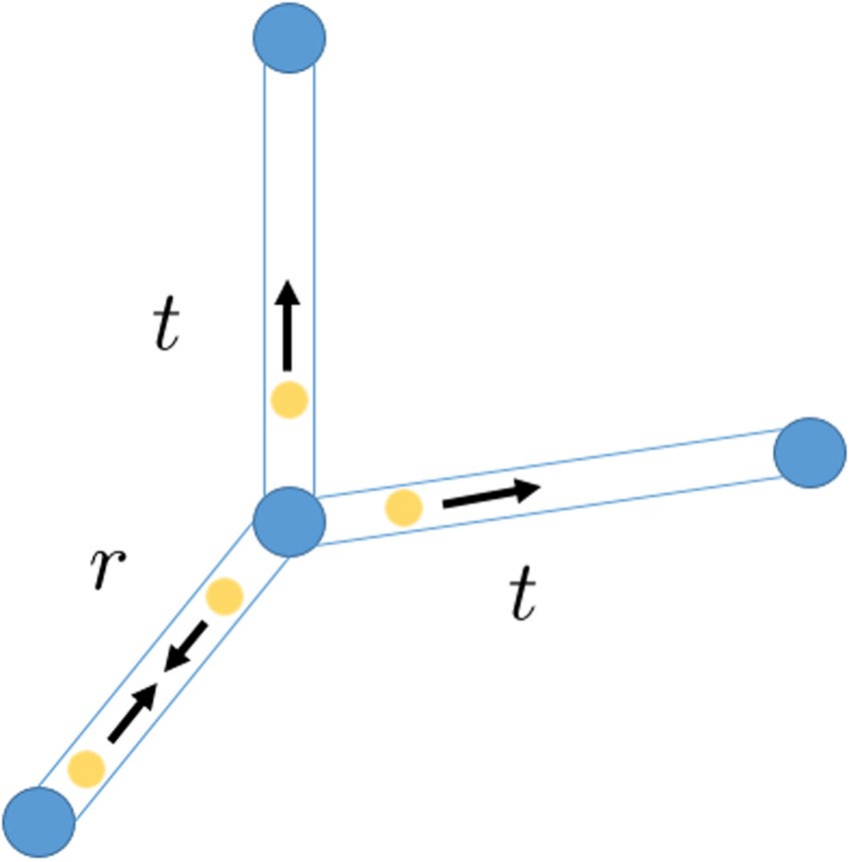
The blue dots are vertices and yellow dots can be regarded as walkers. Together with the black arrow, the yellow dots represent the edge states. This figure can be understood as a photon traveling to a vertex with probability of |*r*|^2^ being reflected back and |*t*|^2^ being transfered to other vertices.

The scattering quantum walk is defined on a graph ${\mathscr{G}}(V,E)$ with *V* being the set of the total vertices and *E* being the set of edges connecting vertices. In this quantum algorithm, the particle walks on the edges of the graph rather than on the vertices. The Hilbert space in this algorithm is defined as

1 $$ {\mathcal H} ={l}^{2}(\{|m,l\rangle \,m,l\in V,ml\in E\}),$$

where state |*m*, *l*〉 is an edge state going from vertex *m* to *l*. The evolution of this quantum walk is defined by the local unitary operator for each vertex. Following the already used notation^18^, we denote Γ(*l*) as the set of vertices connected to vertex *l* and Γ(*l*; *k*) as the set of vertices connected to vertex *l* excluding vertex *k*. The local unitary operator for each vertex is defined as

2 $${U}^{l}|k,l\rangle =-\,{r}^{l}|l,k\rangle +{t}^{l}\,\sum _{v\in {\rm{\Gamma }}(l;k)}\,|l,v\rangle .$$

here *r*^*l*^ and *t*^*l*^ may be different for each vertex. This local operation transforms the state going into the vertex to the state scattering out of the vertex, illustrated in Fig. 1. For simplicity, we call the vertices we want to find as the marked ones and other vertices as the normal ones. Then, for normal vertices, we set

3 $$t=\frac{2}{|{\rm{\Gamma }}(l)|},\,r=1-t$$

and for marked ones

4 $$t=0,\,r=-\,{e}^{i\phi },$$

where |Γ(*l*)| is the number of vertices in the set Γ(*l*). The local unitary operators for the normal one and marked one are denoted as ${U}_{0}^{l}$ and ${U}_{1}^{l}$ respectively.

In search algorithm, oracles are widely used to tell us if the element giving the query is the marked one. In this algorithm, the elements are the vertices of the graph. If we denote the set of marked vertices as ${\mathscr{V}}$ and the set of all vertices as ${\mathscr{N}}$, the oracle can be defined as a function of vertex

5 $$f(x)=\{\begin{array}{ll}1 & x\in {\mathscr{V}}\\ 0 & x\in {\mathscr{N}}\backslash {\mathscr{N}}\end{array}.$$

In this quantum algorithm, a controlled unitary operator works as an oracle, i.e.

6 $${\mathscr{C}}{\hat{V}}_{f}:\,|x\rangle \otimes |m\rangle \mapsto |x\rangle \otimes |m\oplus f(x)\rangle ,$$

where the first one is a state of vertex and the second one is a qubit. When we run this algorithm, the oracle will send back the result according to Eq. (5) and store it in the second qubit. Because ${\mathscr{C}}{\hat{V}}_{f}$ acts on $|x\rangle \otimes |m\rangle$, two extra states, a state of the vertex and a qubit, are necessary to be added to the state of walker for the use of oracle. If we set the state of the walker as |*ψ*_*n*_〉, the state of the whole system is the direct product of these states $|{\psi }_{n}\rangle \otimes |0\rangle \otimes |0\rangle$, before iterating the algorithm. To implement the search, we use a controlled unitary operator ${\mathscr{C}}{\hat{W}}_{1}$ which maps the state |*k*, *l*〉 ⊗ |0〉 ⊗ |0〉 to |*k*, *l*〉 ⊗ |*l*〉 ⊗ |0〉. This step is required for the usage of oracle. Then the controlled unitary operator ${\mathscr{C}}{\hat{V}}_{f}$ will be applied to the state. When the result of the oracle is stored in the qubit, another controlled unitary operator ${\mathscr{C}}{\hat{U}}_{f}^{l}$ will be applied. It will implement local unitary operator ${U}_{f(x)}^{l}$ on the edge states according to the two extra states. After doing this, we will reset the extra states $|l\rangle \otimes |f(l)\rangle$ to $|0\rangle \otimes |0\rangle$ for the next run. This is one iteration of this algorithm which can be explicitly implemented with quantum circuits^18^.

We study the search on the complete graph with *N* vertices. Each vertex is connected with other vertices, so the dimension of the Hilbert space is *N*(*N* − 1) and |Γ(*l*)| = *N* − 1 for any vertex. The initial state of the walker is the equal superposition of all edge states, i.e.

7 $$|{\psi }_{0}\rangle =\frac{1}{\sqrt{N(N-1)}}\,\sum _{a=1}^{N}\,\sum _{b=1,a\ne b}^{N}\,|a,b\rangle .$$

Considering the symmetry of the graph, the quantum walk on *N*(*N* − 1) dimension Hilbert space can be reduced to unitary evolution in a much smaller space consisting of four vectors^18^, such that

8 $$\begin{array}{rcl}|{W}_{1}\rangle  & = & \frac{1}{\sqrt{v(N-v)}}\,\sum _{a=v+1}^{N}\,\sum _{b=1}^{v}\,|a,b\rangle ,\\ |{W}_{2}\rangle  & = & \frac{1}{\sqrt{v(N-v)}}\,\sum _{a=1}^{v}\,\sum _{b=v+1}^{N}\,|a,b\rangle ,\\ |{W}_{3}\rangle  & = & \frac{1}{\sqrt{(N-v)\,(N-v-1)}}\,\sum _{a=v+1}^{N}\,\sum _{b=v+1,a\ne b}^{N}\,|a,b\rangle ,\\ |{W}_{4}\rangle  & = & \frac{1}{\sqrt{v(v-1)}}\,\sum _{a=1}^{v}\,\sum _{b=1,a\ne b}^{v}\,|a,b\rangle .\end{array}$$

here the marked vertices are labeled as 1, 2, …, *v* and normal vertices are labeled as *v* + 1, *v* + 2, …, *N*. These four vectors come from four subspace ${ {\mathcal H} }_{1}$ ${ {\mathcal H} }_{2}$ ${ {\mathcal H} }_{3}$ ${ {\mathcal H} }_{4}$, respectively, where

9 $$\begin{array}{rcl}{ {\mathcal H} }_{1} & = & {l}^{2}(\{|m,l\rangle \,m\in {\mathscr{N}},l\in {\mathscr{V}},ml\in E\}),\\ { {\mathcal H} }_{2} & = & {l}^{2}(\{|m,l\rangle \,m\in {\mathscr{V}},l\in {\mathscr{N}},ml\in E\}),\\ { {\mathcal H} }_{3} & = & {l}^{2}(\{|m,l\rangle \,m,l\in {\mathscr{N}},ml\in E\}),\\ { {\mathcal H} }_{4} & = & {l}^{2}(\{|m,l\rangle \,m,l\in {\mathscr{V}},ml\in E\}).\end{array}$$

Note that if *v* = 1, it will lead to the absence of |*W*_4_〉. It is demonstrated that the efficiency of the algorithm will reach its maximum when the phase shift *φ* is set to *π*^18^. Then when phase shift is set to *π* and $1 < v\ll N$, at any time, the state of the walker |*ψ*_*n*_〉 is

10 $$|{\psi }_{n}\rangle =\frac{1}{2}\,(\begin{array}{c}\sqrt{2}\,\sin (2n+1)\frac{\theta }{2}\\ -\,\sqrt{2}\,\sin (2n-1)\frac{\theta }{2}\\ 2\,\cos \,n\theta \\ 0\end{array}).$$

The components in vector in Eq. (10), from up down, are the amplitudes of vector |*W*_1_〉, |*W*_2_〉, |*W*_3_〉 and |*W*_4_〉, respectively. The vector |*W*_3_〉 is the equal superposition state of the edge states connecting two normal vertices. After measurement, if the walker stands on the edge connected to a marked vertex, a target is found successfully. Thus, the probability of finding a state |*ψ*_*n*_〉 on an edge connected to only normal vertices is |〈*W*_3_|*ψ*_*n*_〉|^2^, which is also the probability that we fail to find the marked vertices. Thus probability of finding the marked vertices is

11 $${P}_{s}=1-|\langle {W}_{3}|{\psi }_{n}\rangle {|}^{2}={\sin }^{2}\,n\theta .$$

Since $n\theta =\frac{\pi }{2}$, *P*_*s*_ = 1, the proper time to measure the walker is $[\frac{\pi }{2}\sqrt{\frac{N}{2v}}]$. When *v* = 1, |*ψ*_*n*_〉 will not have the fourth component, but the evolution of *P*_*s*_ and the proper time to measure the walker will not change.

## Result

### Dynamic of coherence in quantum walk search

Two functions have been proven to be suitable measures of coherence^26^. One is the distance measure based on relative entropy and another one is the *l*_1_ norm, which are denoted as ${C}_{r}(\hat{\rho })$ and ${C}_{l}(\hat{\rho })$, respectively. The explicit expressions of them are

12 $$\begin{array}{rcl}{C}_{r}(\hat{\rho }) & = & S({\hat{\rho }}_{diag})-S(\hat{\rho })=-\,{\rm{Tr}}({\hat{\rho }}_{diag}\,{\mathrm{log}}_{2}\,{\hat{\rho }}_{diag}-\hat{\rho }\,{\mathrm{log}}_{2}\,\hat{\rho }),\\ {C}_{l}(\hat{\rho }) & = & \sum _{i,j,i\ne j}\,|{\rho }_{ij}|,\end{array}$$

where $\hat{\rho }$ is the density matrix of the walker, ${\hat{\rho }}_{diag}$ is the matrix only having the diagonal elements of $\hat{\rho }$ and *ρ*_*ij*_ are the entries of the density matrix. At any time, the density matrix of the walker is

13 $${\hat{\rho }}_{n}=|{\psi }_{n}\rangle \langle {\psi }_{n}|.$$

Note that the state of the walker is a pure state, so the ${C}_{r}(\hat{\rho })$ is reduced to $S({\hat{\rho }}_{diag})$. Applying Eq. (12) to the state of the walker and considering $1 < v\ll N$, we have that

14 $$\begin{array}{rcl}{C}_{r}({\hat{\rho }}_{n}) & = & H\,({\sin }^{2}\,n\theta )+{\cos }^{2}\,n\theta \,{\mathrm{log}}_{2}\,{N}^{2}+{\sin }^{2}\,n\theta \,{\mathrm{log}}_{2}\,2Nv\\  &  & -\,\frac{v(v-1)}{N(N-1)}\,{\mathrm{log}}_{2}\,\frac{1}{N(N-1)}\end{array}$$

and

15 $${C}_{l}({\hat{\rho }}_{n})=2Nv\,{\sin }^{2}\,(n\theta )+{N}^{2}\,{\cos }^{2}\,n\theta +\sqrt{2Nv}N|\sin (2n\theta )|,$$

where *H*(*x*) is the binary Shannon entropy.

**Figure 2:**
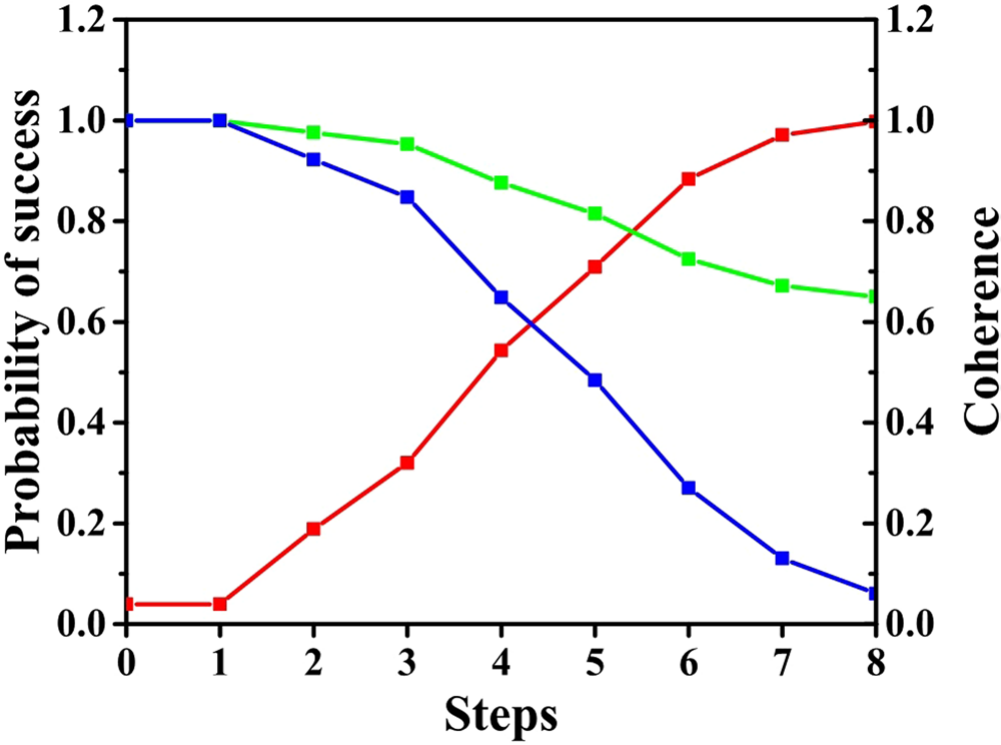
The number of total vertices is 100 and the number of marked vertices is 2. The phase shift is set to *π*. The red line is the probability of success. The green line is the coherence under ${C}_{r}(\hat{\rho })$ and the blue line is the coherence under ${C}_{l}(\hat{\rho })$. The values of two measures of coherence are normalized to 1. The X axis is the iterations of this algorithm. The left Y axis is the probability of success and right Y axis is the value of normalized measure of coherence.

From the above discussions, we can see that coherence and probability of success are both periodic. To reach a high efficiency (evaluated by queries of oracles), it is sensible for us to measure the state before or when probability of success reaches its maximum. So our discussion about probability of success and coherence is confined to the half-period until probability of success reaches its maximum. The first term in ${C}_{r}(\hat{\rho })$ is the binary Shannon entropy which is smaller than 1 and the sum of second term and third term (is) monotonically decreases before the probability of success reaches its maximum. Note that $N\gg v > 1$, the dynamic of coherence is governed by the sum of second term and third term in Eq. (14) under ${C}_{r}(\hat{\rho })$ and second term in Eq. (15) under ${C}_{l}(\hat{\rho })$, so the coherence of the walker will decrease monotonically before the probability of success reaches its maximum. It shows that the walker has consumed the coherence of the initial state to complete the task of search. We define the depletion of coherence from the initial state to the state with maximal probability of success as the consumption of coherence. The connection between the coherence and the probability of success implies that coherence should be viewed as a resource in this algorithm. The results of analysis are also supported by numerical calculation presented in Fig. 2.

For Grover search algorithm, similar result has been obtained recently^30^. Since the quantum algorithm we studied is defined by local unitary operator, it is different from Grover search. For single particle quantum walk search, the state of walker is a single quantum state with multi levels, the methods of quantum entanglement and quantum correlations are in general not applicable, it is expected that the coherence is the resource in this algorithm. If we choose single multi-level quantum state rather than multiple qubits to build quantum database and replace Walsh-Hadamard ${H}^{\otimes n}$ with *U* which transforms |0〉 to $\frac{1}{\sqrt{N}}\,{\sum }_{i=0}^{N-1}\,|i\rangle$, the situation will be the same in Grover search.

### Connection between the efficiency of the algorithm and coherence

**Figure 3:**
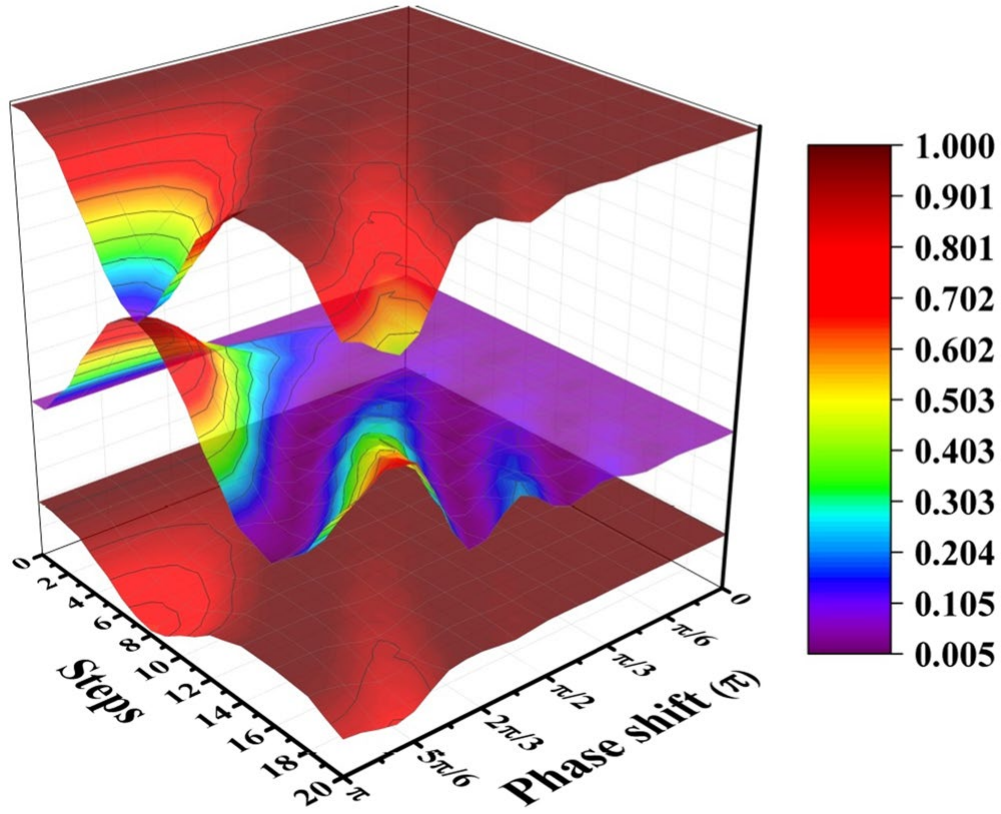
The total number of the vertices is set to 100 and the number of marked vertices is set to 2. The middle surface is the probability of success. The bottom surface and top surface are the coherence under ${C}_{r}(\hat{\rho })$ and ${C}_{l}(\hat{\rho })$, respectively. The values of two measures of coherence are normalized to 1.

**Figure 4:**
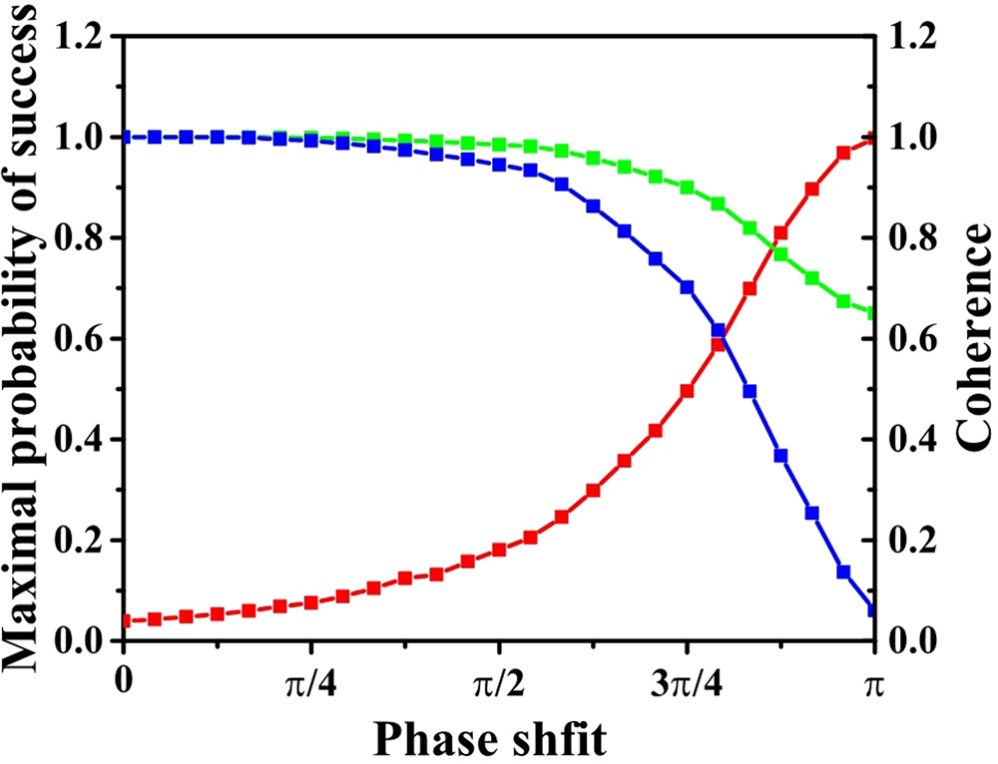
*N* = 100, *v* = 2. The red line is the maximal probability of success. The green line is the coherence under ${C}_{r}(\hat{\rho })$ and the blue line is the coherence under ${C}_{l}(\hat{\rho })$. The values of two measures of coherence are normalized to 1. The X axis is the angle of phase shift. Here we choose 25 values evenly distributed in the interval from 0 to *π*. The left Y axis is the maximal probability of success and right Y axis is the value of normalized measure of coherence.

It has been shown that when *φ* is not *π*, the maximal probability will not be unit, indicating the decrease of the efficiency of the algorithm^18^. We numerically calculated the dynamics of coherence of the processes with different *φ*. When *φ* is substituted by *π* − *φ*, *U* will change into U*. Note that |*ψ*_0_〉 is real, it is easy to see that (U*)^*n*^|*ψ*_0_〉 = (*U*^*n*^|*ψ*_0_〉)*. Then the norm of the amplitude of the state at any time will be the same when *φ* is changed to *π* − *φ*. Further more, the probability of success and coherence will not change. So we only consider *φ* in the region [0,*π*]. The value of coherence and probability of success are presented in Fig. 3. As we can see from the figure, when *φ* is no longer *π*, the correspondence between the success probability and the coherence is preserved. However, the consumption of coherence decreases with lower maximal probability of success. With very low probability of success, the coherence approaches the unit. When phase shift is close to 0, the probability of success is very low, leading to the false of finding targets. In this process, the coherence is stable. For a more clear presentation, we give the mean of local maximal probability of success and corresponding minimal coherence at that step for different *φ* in Fig. 4. In this figure, when maximal probability of success decreases, the minimal coherence increases. This result indicates that when the algorithm is less efficient, the consumption of coherence will decrease.

**Figure 5:**
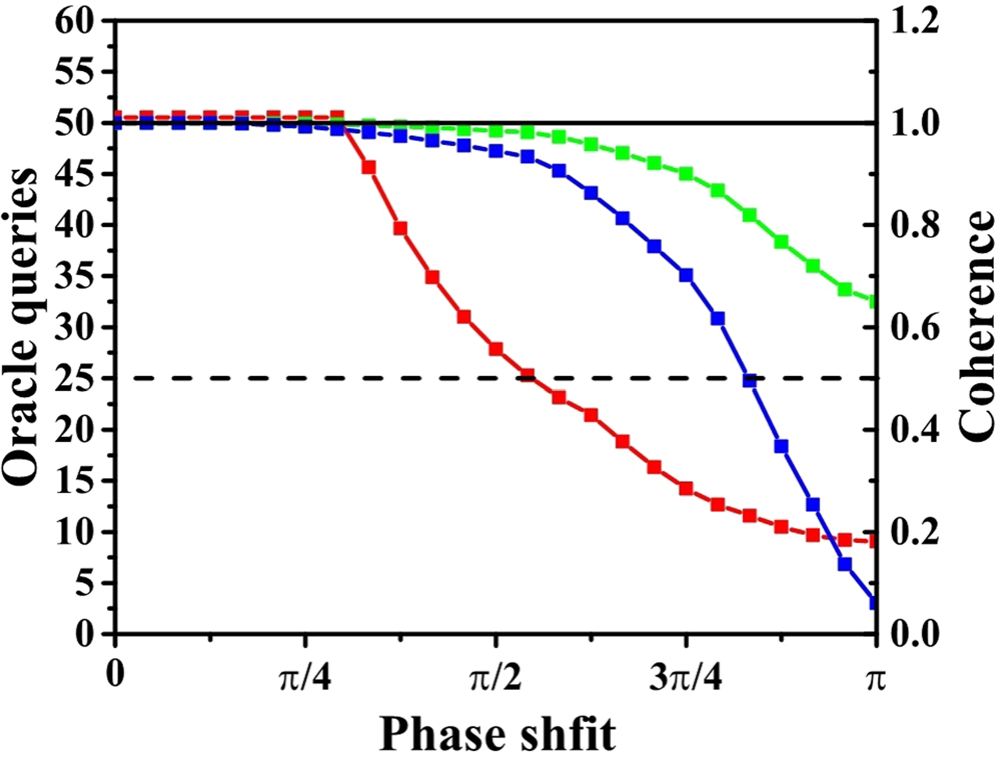
*N* = 100, *v* = 2. The red line is the number of oracle queries. The green line is the coherence under ${C}_{r}(\hat{\rho })$ and the blue line is the coherence under ${C}_{l}(\hat{\rho })$. The values of two measures of coherence are normalized to 1. The X axis is the angle of phase shift from 0 to *π*. The left Y axis is the average number of oracle queries and right Y axis is the value of normalized measure of coherence. The dash black line is the number of oracle queries of classical search with memory and black solid line is the number of oracle queries of classical blind search.

To show the connection between the consumption of coherence and the efficiency of the algorithm more explicitly, the number of oracle queries is chosen to evaluate its efficiency. The number oracle queries can be calculated as follows. Firstly, we will let the particle walk *m* times, each time it will query for an oracle. Then we will measure the particle and reduce the state to one of the edge states. Two oracles will be used to evaluate whether the two vertices connected to the edge are the marked vertices. Suppose we find the marked vertex at the *k*-th run of the algorithm, the number of oracle queries will be *k*(*m* + 2). Since the maximal probability of success is not unit, the marked vertices can not always be found by a single run of the algorithm. Then an average number of oracle queries is defined as

16 $${\bar{Q}}_{\phi ,m}=\sum _{k=1}^{\infty }\,{(1-{P}_{\phi }(m))}^{k-1}\,{P}_{\phi }(m)k(m+1)=\frac{m+2}{{P}_{\phi }(m)},$$

where *P*_*φ*_(*m*) is the probability of success when we measure the walker at *m*-th step. Here, *m* is chosen to minimize ${\bar{Q}}_{\phi ,m}$. This quantity represents the efficiency of the algorithm. With fewer oracle queries, the algorithm will be more efficient. The result for the case of 100 vertices with 2 marked vertices is plotted in Fig. 5. It shows that the classical blind search provides a lower bound for the efficiency of the quantum search algorithm. The algorithm will be less efficient than the classical search with memory, if the consumption of coherence is below a proper value. This is similar to that the decoherence in quantum walk can cause a transition from the quantum walk to classical walk, which can be directly shown by the stochastic quantum walk^33^. However, decoherence comes from noises, which is taken into account later, in the realistic implementation and there is no noise in our consideration now. These results tell us that coherence is responsible for the speed-up of this quantum search algorithm.

### Walk with no coherence

In this section, we focus on the probability of success when the initial state is incoherent. An incoherent initial state^26^ should be expressed as

17 $${\rho }_{0}=\sum _{a=1}^{N}\,\sum _{b=1,a\ne b}^{N}\,{P}_{ab}|a,b\rangle \langle a,b|,$$

where ${\sum }_{a,b}\,{P}_{ab}=1$. At any time, density matrix of the system is

18 $$\rho (n)={U}^{n}{\rho }_{0}{U}^{\dagger n}.$$

This can also be viewed as that *U* is performed *n* times on ensemble {|*a*, *b*〉} with probability *P*_*ab*_ for the state |*a*, *b*〉. With different states in the ensemble as initial state, the probability of success for state |*a*, *b*〉 is

19 $${P}_{s}(|a,b\rangle ,n)=1-\sum _{k=v+1}^{N}\,\sum _{l=v+1,l\ne v}^{N}\,|\langle k,l|{U}^{n}|a,b\rangle {|}^{2}.$$

The success probability for an initial incoherent state would be

20 $${P}_{s}(n)=\sum _{a=1}^{N}\,\sum _{b=1,a\ne b}^{N}\,{P}_{s}(|a,b\rangle ,n){P}_{ab}.$$

The Eq. (19) shows that, if the states come from the same subspace, they will share the same value of *P*_*s*_(|*a*, *b*〉, *n*). Then we define ${P}_{s}({ {\mathcal H} }_{i},n)$, which is the probability of success with an initial state $|a,b\rangle \in { {\mathcal H} }_{i}$. As we stated above, there are four different subspaces (number of marked ones is more than 1). In the case of an incoherent initial state, the probability of success can be reformulated as

21 $${P}_{s}(n)=\sum _{i=1}^{4}\,{a}_{i}{P}_{s}({ {\mathcal H} }_{i},n),$$

where

22 $${a}_{i}=\sum _{|a,b\rangle \in { {\mathcal H} }_{i}}\,{P}_{ab}.$$

**Figure 6:**
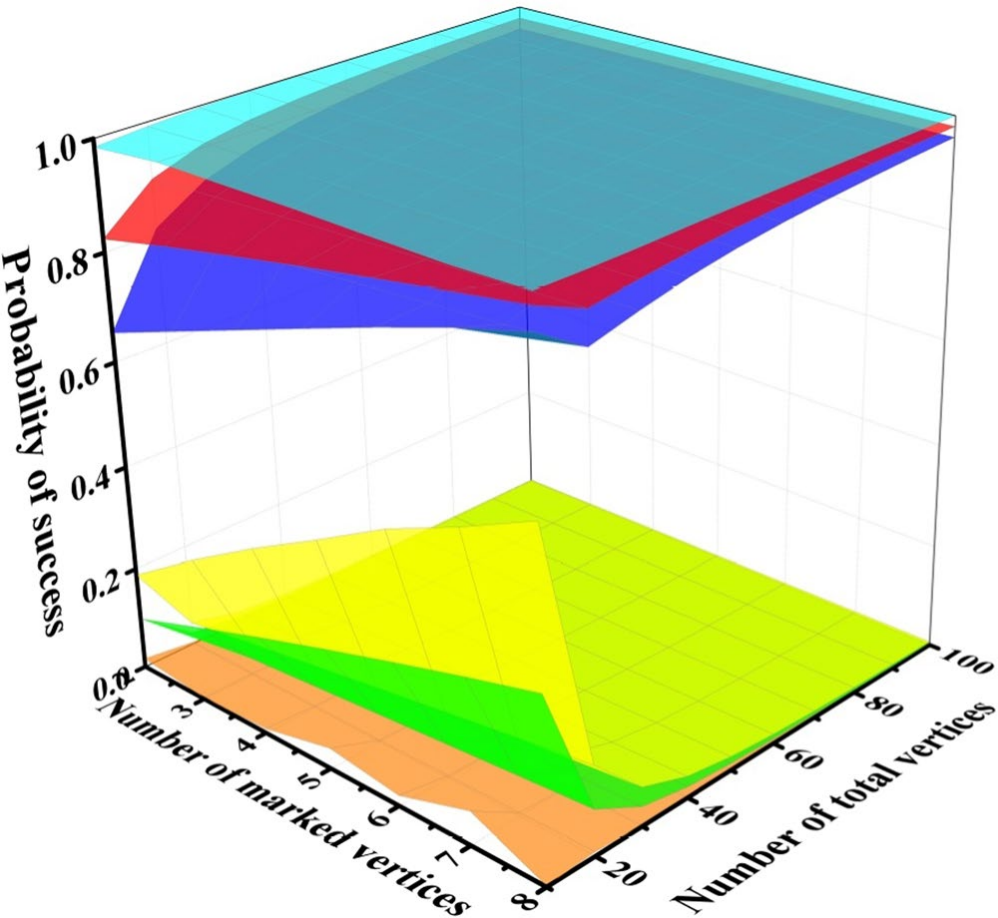
The red surface and green surface are the means of ${P}_{s}({ {\mathcal H} }_{2},n)$ and ${P}_{s}({ {\mathcal H} }_{3},n)$, respectively. The surfaces above and below the red and green surface are the maximal and minimal values of ${P}_{s}({ {\mathcal H} }_{2},n)$ and ${P}_{s}({ {\mathcal H} }_{3},n)$ respectively with different *N* and *v* in 20 steps. The Z axis is the probability of success.

Note that at the beginning, ${P}_{s}({ {\mathcal H} }_{3},0)=0$, it is easy to obtain that *a*_1_ + *a*_2_ + *a*_4_ = *P*_*s*_(0). For a state from the subspace ${ {\mathcal H} }_{4}$, it will transform between |*a*, *b*〉 and |*b*, *a*〉 with additional negative sign with implementation of *U*. Thus ${P}_{s}({ {\mathcal H} }_{4},n)$ will be 1 all the time. When we apply *U* on the state from ${ {\mathcal H} }_{1}$, it will turn into one from ${ {\mathcal H} }_{2}$ with additional phase shift *π*. Thus for the states from subspace ${ {\mathcal H} }_{1}$ and ${ {\mathcal H} }_{2}$, it is easy to obtain that ${P}_{s}({ {\mathcal H} }_{2},n)={P}_{s}({ {\mathcal H} }_{1},n+1)$ with ${P}_{s}({ {\mathcal H} }_{2},0)=1$. We numerically calculate *P*_*s*_(*n*) for states from subspace ${ {\mathcal H} }_{2}$ and ${ {\mathcal H} }_{3}$ and present the result in Fig. 6.

Then we can conclude that when $N\gg v$, ${P}_{s}({ {\mathcal H} }_{1},n)$, ${P}_{s}({ {\mathcal H} }_{2},n)$ will converge to one and ${P}_{s}({ {\mathcal H} }_{3},n)$ will be zero. This result is very reasonable. In another scheme of quantum search, quantum amplitude amplification, if *A*|0〉 is totally projected on the good subspace or bad subspace, the probability of success will be 1 or 0, respectively^34^. However, this only holds for $N\gg v$ in quantum scattering walk search. Recall that ${P}_{s}({ {\mathcal H} }_{4},n)$ is 1 all the time, the probability of success will be

23 $${P}_{s}(n)={a}_{1}+{a}_{2}+{a}_{4}={P}_{s}(0).$$

The result above implies that the probability of success is only determined by the initial state *ρ*_0_. It means that the scattering quantum walk search totally loses its power on all incoherent initial states and further shows that coherence should be considered as a resource in this algorithm.

### Experimental realization of scattering quantum walk search

The scattering quantum walk was initially proposed to realize the quantum walk using linear optical elements. Any *U*(*N*) operator can, in principle, be decomposed into sequence of *SU*(2) operators which describe the behaviors of linear optical elements, such as beam splitters and phase shifters^35,36^. Thus the local unitary operators used in the algorithm can be effectively implemented by linear optical elements via constructing optical networks. Because an optical network has *N* inputs and outputs, it is also called an optical multiports.

In the implementation of scattering quantum walk, the vertex can be represented by the multiports^37^. For example, a vertex with two edges corresponds to a multiports with two inputs and outputs. The states of inputs represent the edge states scattering into the vertex and the outputs are the edge state scattering out. The choice of the multiports depends on the topology considered in the question. In our work, the complete graph is studied. For a complete graph with *N* vertices, *N* multiports with *N* − 1 inputs and outputs are needed in the iteration of the algorithm.

**Figure 7:**
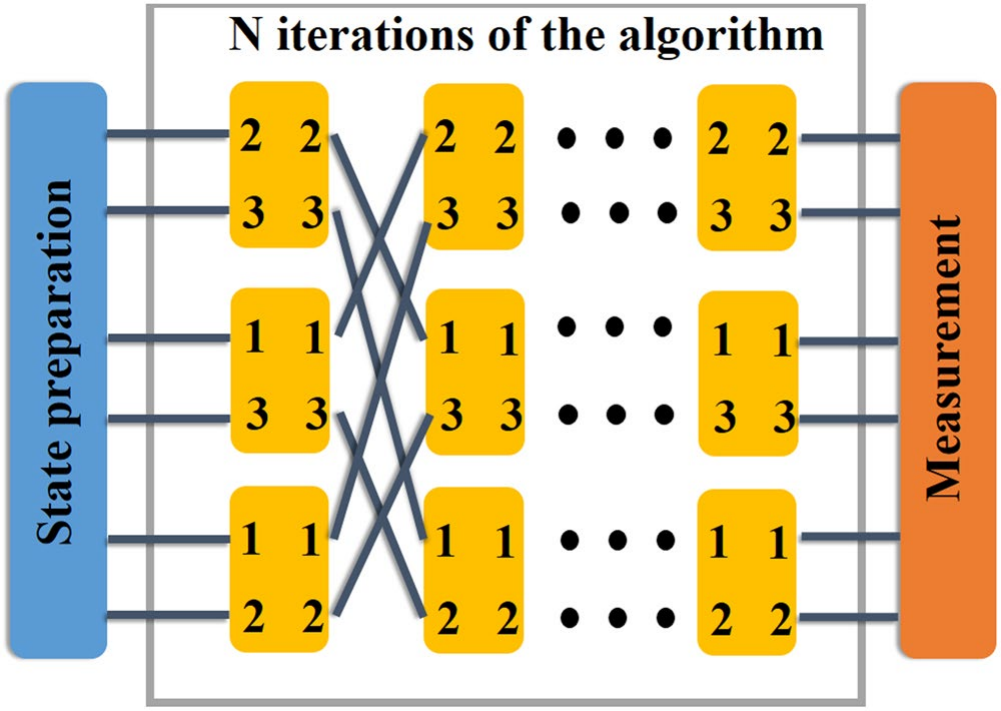
This illustrates the set of experiment for three vertices complete graph. The blue block and orange block are the instruments used to prepare the state and measure it. In the middle part, it represents the iterations of the algorithm. Each yellow block is a multiports with two inputs and outputs. In each column, from up to down, they are vertex 1, 2 and 3, respectively. And each column is an iteration of the algorithm. There are numbers labeled on the yellow blocks which represents different ports. The numbers labeled on the left sides of the yellow block give which vertices should be connected to these ports and those on the right side decide which vertices these ports should connect to. All the multiports are yellow, which says that they are homogeneous, implementing the same local unitary operators. To carry out the search algorithm, we need to mark the target vertices. If vertex 1 is marked, the first row of multiports should be colored accordinglly rather than yellow, representing the different local unitary operations performed on them.

A complete set of the experiment of the scattering quantum walk is shown below. First single photons are generated and sent into the preparation process to be transformed to the desired state. Then the photon enters the process of the algorithm. In this part, *N* multiports are put into an array and *n* such arrays are arranged in a line. The multiports are designed according to the behavior of the local unitary operator for each vertex in the scattering quantum walk on the complete graph. By the time the single photons have been through one of the array, one iteration of the algorithm has been done. At the end, the measurement instrument is placed to detect the edge the photon standing on. By changing the number of arrays, we can set the iterations of the algorithm making the measurement of walker at any times possible via placing the measurement instrument at the end. Given the complete graph with three vertices, an illustration of the realization is presented in Fig. 7.

To implement a *U*(*N*) operator, *O*(*N*^2^) discrete linear optical elements are required. It is extremely cumbersome to set and align the optical networks making the realization almost impossible. The integrated waveguides can be used to simplify the construction of the optical networks^38,39^. The optical networks can be entirely integrated onto a chip to be more optically stable. The integrated photonic chip has been widely used in the implementation of quantum walk, boson sampling, quantum circuit^12,40,41^. Then it will be possible to check our result in the realization of the algorithm utilizing the integrated waveguides.

### The effect of noises on the performance of the algorithm

Without regard for noise, we have given the connection between the coherence of system and success probability or the efficiency of the algorithm. However, a realistic quantum system must bear some type of noises. To check the role of coherence with noise in the system, two types of noises, photon loss and random phase shift, are considered for the realization of the algorithm using optical multiports. This consideration has already been taken in the study of effect of errors in quantum walk search on *n* dimensional hypercube^37^.

Photon loss is a common noise in optical networks, coming from the imperfection of multiports and the scattering and absorption during the propagation between the multiports. The effect of the photon loss is characterized by the linear loss rate *η* depending on the arms of multiports. The operator *D* is defined to describe the state of walker suffering from the photon noise, such as

24 $$D=\sum _{a=1}^{N}\,\sum _{b=1,a\ne b}^{N}\,{\eta }_{a,b}|a,b\rangle \langle a,b|.$$

Suppose the original density matrix of the walker is *ρ*, the state of walker involving the photon loss is

25 $$\rho ^{\prime} +[1-tr(\rho ^{\prime} )]|0\rangle \langle 0|,$$

where *ρ*′ = *Dρ* and |0〉 is the vacuum state. It says that if the state is |*a*, *b*〉, then it may become a vacuum state, indicating the photon loss, with probability $1-{\eta }_{a,b}^{2}$. Let us first consider a uniform distribution that *η*_*a*,*b*_ = *η* for any *a* and *b* at any time. The density matrix of the state with uniform linear loss rate is

26 $$\rho (t)={\eta }^{2t}|\psi (t)\rangle \langle \psi (t)|+(1-{\eta }^{2t})|0\rangle \langle 0|,$$

where |*ψ*(*t*)〉 is the state in the perfect optical networks. When the walker is measured at the t-th step, the success probability is the original one multiplied by *η*^2*t*^. Since *η* < 1, the probability of finding the walker on the target state is extremely small in the limit of long iterations. The coherence of the system can be easily calculated according to Eqs (14) and (15), such that

27 $$\begin{array}{rcl}{C}_{r}(\rho (t)) & = & {\eta }^{2t}{C}_{r}(|\psi (t)\rangle )-(1-{\eta }^{2t})\,{\mathrm{log}}_{2}\,(1-{\eta }^{2t})\\ {C}_{l}(\rho (t)) & = & {\eta }^{2t}{C}_{l}(|\psi (t)\rangle ).\end{array}$$

**Figure 8:**
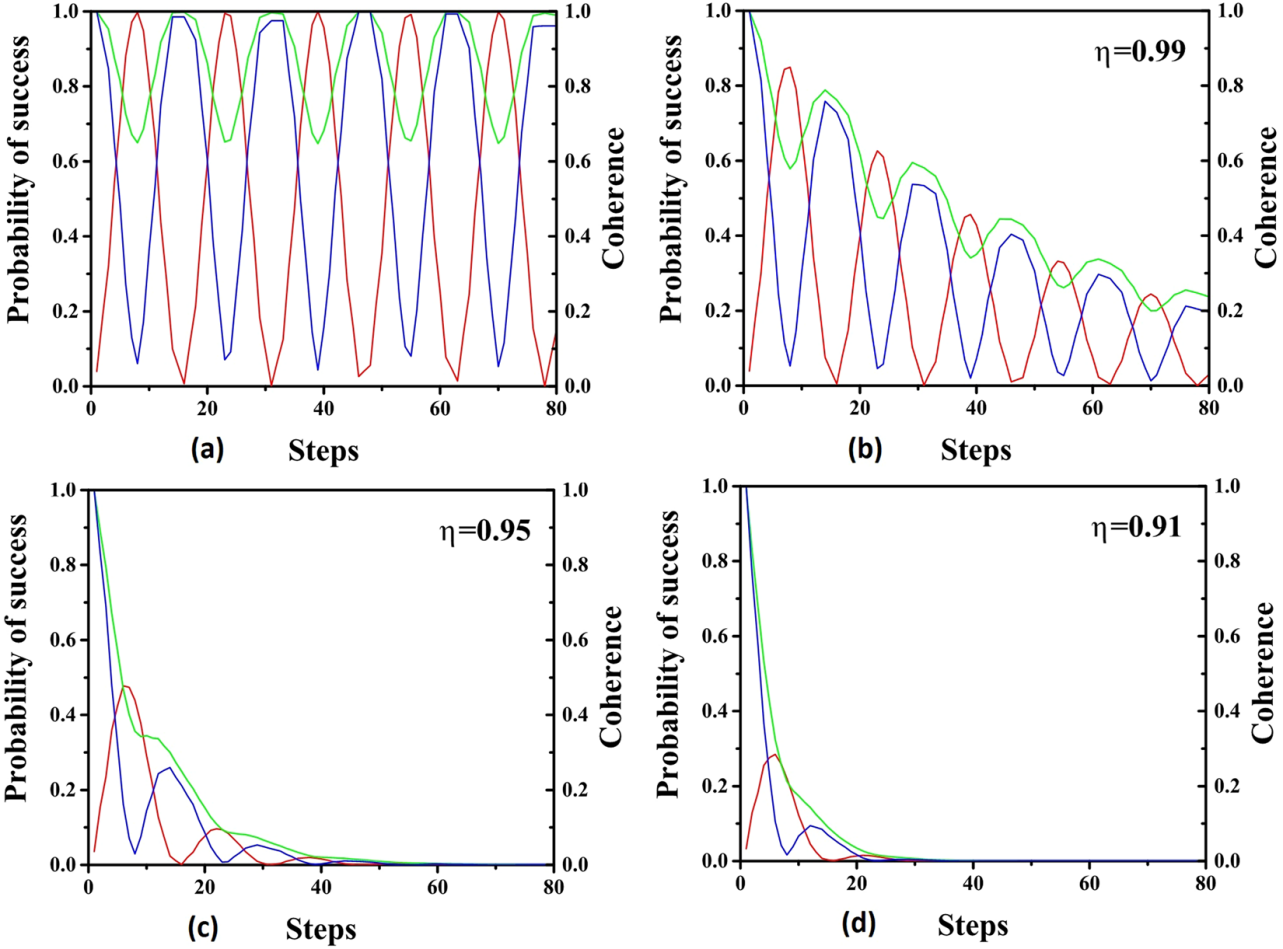
Here the total vertices of the complete graph is 100 and there are 2 marked vertices. The red line is the probability of success. The green line is the coherence under ${C}_{r}(\hat{\rho })$ and the blue line is the coherence under ${C}_{l}(\hat{\rho })$. The values of two measures of coherence are normalized to 1. The liner loss rates are set as (**a**) 1 (**b**) 0.99 (**c**) 0.95 (**d**) 0.91.

From above, we see that the original expression of coherence are both multiplied by *η*^2*t*^ in *C*_*r*_(*ρ*(*t*)) and *C*_*l*_(*ρ*(*t*)), but there is an additional term −(1 − *η*^2*t*^) log_2_(1 − *η*^2*t*^) in *C*_*r*_(*ρ*(*t*)) compared with the *C*_*l*_(*ρ*(*t*)). The success probability and coherence under *C*_*r*_(*ρ*) and *C*_*l*_(*ρ*) are presented in the Fig. 8 with varied linear loss rate. Since the success probability is multiplied by a factor *η*^2*t*^ with *η* < 1, it will undergo an oscillatory decay, which is shown in the figure. For a fixed *η*, the success probability is approaching to zero after a large number of iterations of algorithm. With higher probability of photon loss, the smaller value of *η*, the decay of success probability is in the faster pace. The akin phenomena can be observed for the coherence in the system under both measurement, indicating the leaking of coherence due to the photon losses. Because the operation of algorithm and the photon loss both contribute to the consumption of coherence, we can not always anticipate the validation of relation between the consumption of coherence and the increase of success probability. When the photon losses exit, the algorithm still still try to improve the success probability by consuming the coherence. However, the photon losses leak the coherence of the system leaving less coherence for the operation of the algorithm resulting in the low success probability which further shows that the essential role of coherence in the scattering quantum walk search.

Another noise taken into consideration is the random phase shifts. It arises from the stochastic changes of the designated optical path length which is the combination of thermal noise and imperfection of linear optical networks. The effect of this noise can be characterized by an operator *R*. After each iteration of the algorithm, the effect of random phase shift is equivalent to the application of the operator *R*. For the edge state |*a*, *b*〉, performance of operator *R* will give

28 $$R|a,b\rangle ={e}^{i{\varphi }_{a,b}}|a,b\rangle .$$

here, *ϕ*_*a*,*b*_ is a random phase which is different for distinctive edge states and changes at each iteration. The random phase shift will generate a distribution, {*ϕ*_*a*,*b*_}. Owing to the allowance of global phase factor in quantum mechanics, the average value of distribution can always be set as 0.

**Figure 9:**
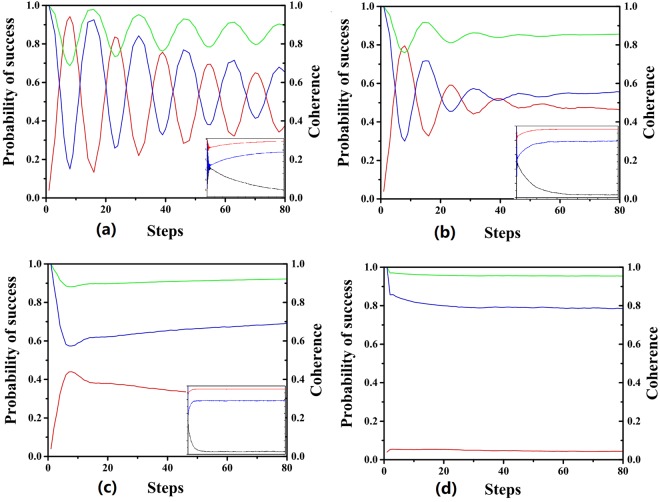
Here the total vertices of the complete graph is 100 and there are 2 marked vertices. The red line is the probability of success. The green line is the coherence under ${C}_{r}(\hat{\rho })$ and the blue line is the coherence under ${C}_{l}(\hat{\rho })$. The values of two measures of coherence are normalized to 1. The {*ϕ*_*a*,*b*_} is subjected to Gaussian distribution centered at zero. The variances are set to (**a**) *π*/24 (**b**) *π*/12 (**c**) *π*/6 (**d**) *π*/2.

Given a special case, the coherence and success probability are numerically calculated and presented in Fig. 9. When the random phase shifts are slight, the success probability and coherence oscillate with decreasing amplitude. With larger variance, they decrease in faster pace due to the stronger effect of random phase shifts. For even larger variance, the oscillations are negligible. After a long run, the value of success probabilities are close to the initial ones. Note that the coherence is invariant after the application of random phase shifts, which differs from the case of photon loss. Thus we can see that the connection between the coherence and the success probability is still valid. However, with the random phase shifts, the success probabilities approach to initial values in different paces depending on the variance of the distributions and a larger variance will result in a faster pace. It can be concluded that the random phase shift prevents the algorithm from consuming the coherence and with larger variance the fewer coherence can be used by the algorithm leading to the low success probability, which shows the essential role of coherence. This also reveals that coherence is responsible for the acceleration of the algorithm because the algorithm losses its power when consumption of coherence is hindered by the random phase shifts.

## Discussion

In this paper, we calculate the coherence in the scattering quantum walk search algorithm on complete graph and associate it with the probability of success under the condition $N\gg v > 1$. We found that the coherence of the system decreases while the probability of success increases until reaching its maximum. It shows that coherence of the system is consumed to complete the task of search. Since the system we considered is a single quantum state with multiple levels, the methods of quantum entanglement and quantum correlations are in general not applicable, the coherence clearly can be considered as the resource in this algorithm. Besides, with the value of phase shift varied, the decrease of maximal success probability causes the simultaneous decline of coherence consumption, which gives rise to the potential correspondence between the efficiency of the algorithm and the consumption of coherence. Choosing oracle queries to evaluate the efficiency of the algorithm, we find the consumption of coherence is an monotonically increasing function of the efficiency of algorithm. If the coherence consumption is smaller than a given value, the quantum algorithm we investigated will be less efficient than the classical search with memory. Without coherence consumption, the quantum walk search algorithm will have the same efficiency as the classical blind search. That is to say, the coherence is responsible for the speed-up of this quantum algorithm. We also consider the probability of success of this algorithm starting with an incoherent state and discover that it keeps unchanged compared with the initial time. Last but not the least, the photon loss and random phase shifts are studied to see the impact of noise on the performance of the algorithm. The photon loss causes the decrease of coherence, giving smaller success probability. The random phase shifts prevent the algorithm from consuming the coherence leading to low success probability. It clearly shows that the coherence is a key resource in this algorithm.

Decoherence is a hot topic in the study of quantum walk and a lot of work has focused on it. In terms of position variance, the transition between the quantum walk and classical random walk exists by considering decoherence of the coin^42^. The effect of noise is considered in the construction and a direct transition between quantum walk and classical random walk can be observed by changing the relative rate of the unitary and nonunitary processes^33^. In these works, the rate of decoherence is associated with the performance of the quantum walk and with more decoherence the algorithm will have worse performance, indicating the key role of coherence in quantum walk. It is similar to the results of our work that the more coherence in the system will lead to better performance of the algorithm. However, the decoherence caused by the noises does not always cause unsatisfactory performance. In some quantum walk tasks, it enhances the performance such as noise-assisted quantum walk in which the transport efficiency is improved^43–45^. This shows us that how coherence affects the performance of the algorithm may vary in different quantum walks and further researches should be done in other quantum walk tasks.

Our work shows that coherence plays an essential role and is responsible for the speed-up in scattering quantum walk search on complete graph. We believe that our method can be generalized to other quantum walk algorithms and may have applications in the quantum computation processing.

## Method

### Detailed analysis of the scattering quantum walk search on complete graph

Since the quantum walk is implemented on *N*(*N* − 1) edge states, which consists of a very large Hilbert space, hindering our analysis. In original work^18^, the analysis of the evolution of the scattering quantum walk search on complete graph is done by considering the symmetry of the graph. The group of automorphisms of the graph is denoted as ${\mathscr{A}}$. A element of the group ${\mathscr{A}}$, *a* is a mapping which maps two connected vertices to another two connected vertices, such that {*v*_1_, *v*_2_} is mapped to {*a*(*v*_1_), *a*(*v*_2_)}. The effect of an automorphism is equivalent to a unitary operator *U*_*a*_ on the edge states. It transforms |*v*_1_, *v*_2_〉 to |*a*(*v*_1_), *a*(*v*_2_)〉. In our work, elements of ${\mathscr{A}}$ are specific to leave the marked vertices fixed. The Hilbert space of the quantum walk can be decomposed into four subspaces, i.e. ${\mathcal H} ={\oplus }_{j=1}^{4}\,{ {\mathcal H} }_{j}$ and ${ {\mathcal H} }_{j}$ are presented in Eq. (9). These subspaces are the smallest invariant subspaces under all automorphisms. The four vectors in Eq. (8) are invariant under the operations of all automorphisms, which are the only ones in corresponding subspaces. Suppose the subspaces these four vectors consisted of are ${\mathscr{S}}={l}^{2}\{|{W}_{j}\rangle |j=1,2,3,4\}$, any state |*ψ*〉 belonging to ${\mathscr{S}}$ satisfies that *U*_*a*_|*ψ*〉 = |*ψ*〉 for any *a*. It can be proved that [*U*, *U*_*a*_] = 0 is satisfied for all *a*. Then it is easy to obtain that *U*_*a*_*U*|*ψ*〉 = *U*|*ψ*〉 for all *a*. This tells us that if a state |*ψ*〉 is from *S* then *U*|*ψ*〉 is also in that subspace. Since the initial state can be reformulated as

29 $$|{\psi }_{0}\rangle =\,\sqrt{\tfrac{v(N-v)}{N(N-1)}}(|{W}_{1}\rangle +|{W}_{2}\rangle )+\sqrt{\tfrac{(N-v)\,(N-v-1)}{N(N-1)}}|{W}_{3}\rangle +\sqrt{\tfrac{v(v-1)}{N(N-1)}}|{W}_{4}\rangle ,$$

the evolution of the quantum walk can be described in the subspace. The unitary operator of the walker can be represented as

30 $$U=(\begin{array}{cccc}0 & q & s & 0\\ {e}^{i\phi } & 0 & 0 & 0\\ 0 & s & -q & 0\\ 0 & 0 & 0 & {e}^{i\phi }\end{array}),$$

where

31 $$q=-\,r+(v-1)\,t=-\,1+\frac{2v}{N-1},\,s=\sqrt{1-{q}^{2}}=t\sqrt{v(N-v-1)}.$$

This is a four dimensional unitary operator, we can easily see the evolution induced by this operator via calculating the eigenvalues and eigenvectors. Recall that the phase shift is chosen to be *π* to achieve the best performance. In this case, at any time the state of the walker is

32 $$|{\psi }_{n}\rangle =D(\begin{array}{c}\sqrt{2(N-1)}\,\sin (2n+1)\frac{\theta }{2}+{(-1)}^{n}A\\ -\,\sqrt{2(N-1)}\,\sin (2n-1)\frac{\theta }{2}+{(-1)}^{n}A\\ 2\sqrt{(N-v-1)}\,\cos \,n\theta -{(-1)}^{n}B\\ {(-1)}^{n}C\end{array}),$$

where

33 $$\begin{array}{ccc}A & = & \sqrt{\tfrac{v{(N-v-1)}^{2}}{{(N-1)}^{2}}},\\ B & = & \sqrt{\tfrac{{v}^{2}(N-v-1)}{{(N-1)}^{2}}},\\ C & = & \sqrt{\tfrac{v(v-1)\,{(2N-v-2)}^{2}}{(N-v)\,{(N-1)}^{2}}},\\ D & = & \sqrt{\tfrac{(N-v)\,(N-1)}{{(2N-v-2)}^{2}N}},\\ \tan \,\theta  & = & \tfrac{\sqrt{v(2N-v-2)}}{N-v-1}.\end{array}$$

If the condition $N\gg v$ is satisfied, we can recover the Eq. (10).
